# Progress of Ferroptosis in Ischemic Stroke and Therapeutic Targets

**DOI:** 10.1007/s10571-024-01457-6

**Published:** 2024-02-23

**Authors:** Xinjuan Tian, Xiang Li, Mengtian Pan, Lele Zixin Yang, Yunman Li, Weirong Fang

**Affiliations:** 1 School of Basic Medical Sciences and Clinical Pharmacy, China Pharmaceutical University, Mailbox 207, Tongjiaxiang 24, Nanjing, Jiangsu 210009 People’s Republic of China; 2 The Pennsylvania State University, State College, PA 16801 USA

**Keywords:** Ischemic stroke, Ferroptosis, Iron overload, Lipid peroxidation

## Abstract

Ferroptosis is an iron-dependent form of programmed cell death (PCD) and ischemic stroke (IS) has been confirmed to be closely related to ferroptosis. The mechanisms of ferroptosis were summarized into three interrelated aspects: iron metabolism, lipid peroxide metabolism, as well as glutathione and amino acid metabolism. What’s more, the causal relationship between ferroptosis and IS has been elucidated by several processes. The disruption of the blood–brain barrier, the release of excitatory amino acids, and the inflammatory response after ischemic stroke all lead to the disorder of iron metabolism and the antioxidant system. Based on these statements, we reviewed the reported effects of compounds and drugs treating IS by modulating key molecules in ferroptosis. Through detailed analysis of the roles of these key molecules, we have also more clearly demonstrated the essential effect of ferroptosis in the occurrence of IS so as to provide new targets and ideas for the therapeutic targets of IS.

**Graphical Abstract:**

Three abnormal cell metabolism pathways contribute to ferroptosis after ischemic stroke, and many key regulatory compounds in ferroptosis can play important therapeutic roles.

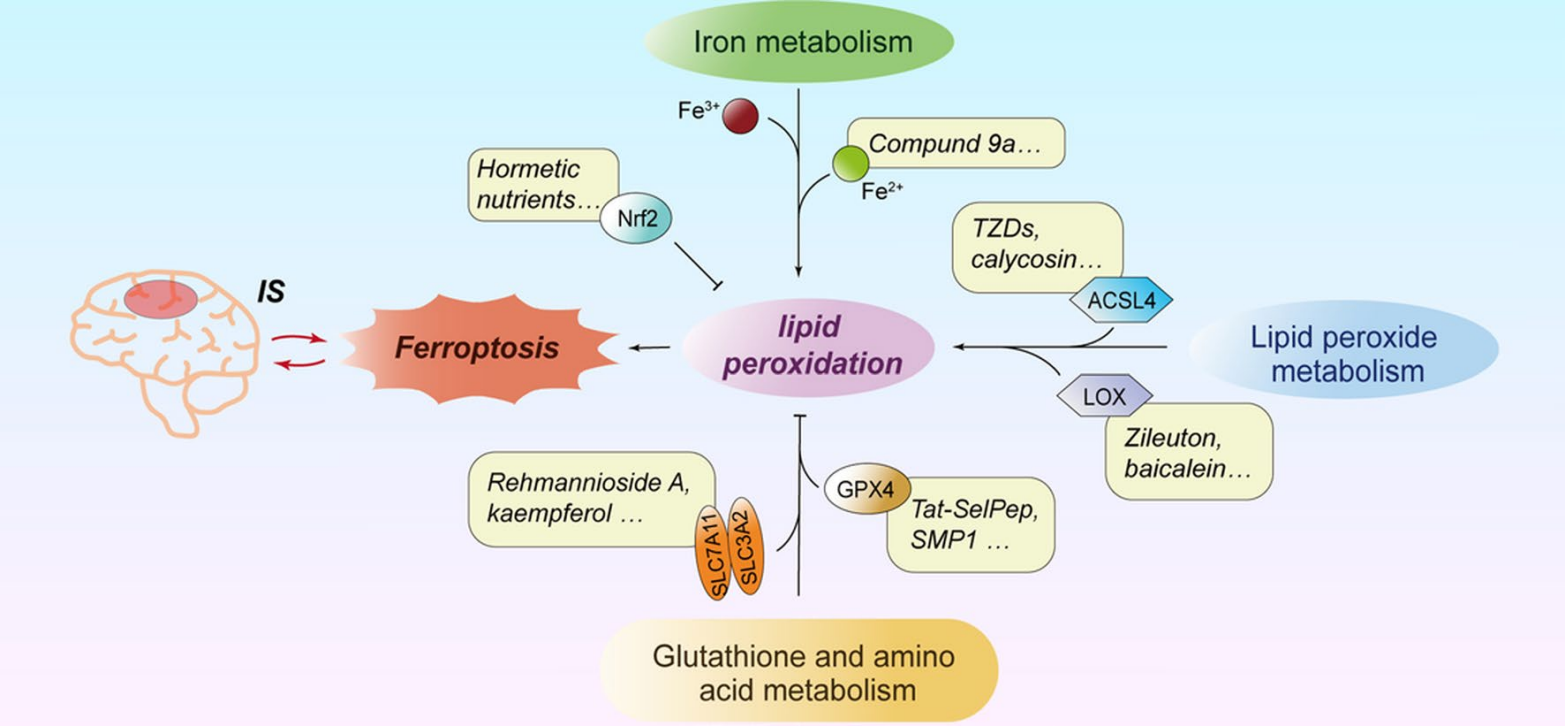

## Introduction

Stroke is a clinically common cerebrovascular disease, which is the first cause of death and the main cause of disability in the world. Because of its high incidence, mortality, disability rate, recurrence and medical costs, stroke has emerged as a serious health problem of general concern. Stroke is divided into ischemic stroke (IS), hemorrhagic stroke (HS), and subarachnoid hemorrhage (SAH), of which IS accounts for 87% of all occurrences (Ajoolabady et al. 2021). Due to cerebral ischemia or hemorrhage, the normal blood supply to neurons is destroyed, thus promoting a series of pathophysiological reactions, and finally leading to neuronal death. This process involves the interaction of many mechanisms, including excitatory amino acid toxicity, free radical release, neuronal apoptosis, necrosis, autophagy, over-activation of inflammatory response (Qin, et al. 2022).

Ferroptosis is involved in many central nervous system (CNS) diseases, such as stroke, Alzheimer’s disease, Parkinson’s disease, Huntington’s chorea (Yuan et al. 2021a). In 2003, the small molecular compound erastin, which can initiate the nonapoptotic cell death process was first discovered and named (Dolma et al. 2003). In 2008, Yang and Stockwell named two small molecules (RSL3 and RSL5) based on a synthetic lethal screening system, which can activate iron-dependent and non-apoptotic cell death in the presence of oncogenic RAS (Yang and Stockwell 2008). In the same year, Conrad et al. also demonstrated a new type of cell death mediated by the loss of glutathione peroxidase 4 (GPX4) (Seiler et al. 2008). However, it wasn’t until 2012, that Brent R Stockwell et al. finally termed this unique iron-dependent form of nonapoptotic cell death “ferroptosis,” in that iron is crucially important during this process (Dixon et al. 2012). In short, the feature (above effect) of ferroptosis lies in the fact that the iron-dependent accumulation of lipid hydroperoxides reaches lethal levels (Stockwell et al. 2017). The cellular morphology of ferroptosis is mainly characterized by reduced mitochondrial size, condensed mitochondrial membrane density, decreased or vanishing mitochondrial cristae, and rupture of the outer mitochondrial membrane (Yang et al. 2021), which are very different from other cell death modes, including apoptosis, necrosis, and autophagy (Jin et al. 2021).

With the advent of ferroptosis as a defined process, a large number of investigations have focused on exploring the correlation between IS and this death mode. Considerable experimental results supported the conclusion that the content of iron, lipid peroxidation (LPO) and ferritin (FT) was elevated in the brain damaged region of rats after middle cerebral artery occlusion (MCAO) (Liu et al. 2022). This phenomenon was also observed in recent, high-fidelity *in-situ* imaging using a H_2_S triggered and H_2_S releasing near-infrared fluorescence showed that the MCAO could induce ferroptosis (Liang et al. 2022).

On this basis, we not only summarized the effect of ferroptosis in the pathophysiological process of IS, but also listed the corresponding drug targets and potential compounds, aiming to provide new therapeutic ideas for reducing post-stroke injury by targeting ferroptosis.

## Ferroptosis in Ischemic Stroke

### Cellular Metabolic Mechanism of Ferroptosis

Ferroptosis can be initiated through two major pathways: the extrinsic, or transporter-dependent pathway, and the intrinsic, or enzyme regulated pathway. The extrinsic pathway can be triggered by inhibiting membrane transporters, such as cystine/glutamate transporters (system Xc^−^), or activating ferroportin (FPN), transferrin (TF), and lactotransferrin (LTF). The activation of the intrinsic pathway is achieved by blocking antioxidant enzymes like GPX4.

**Fig. 1:**
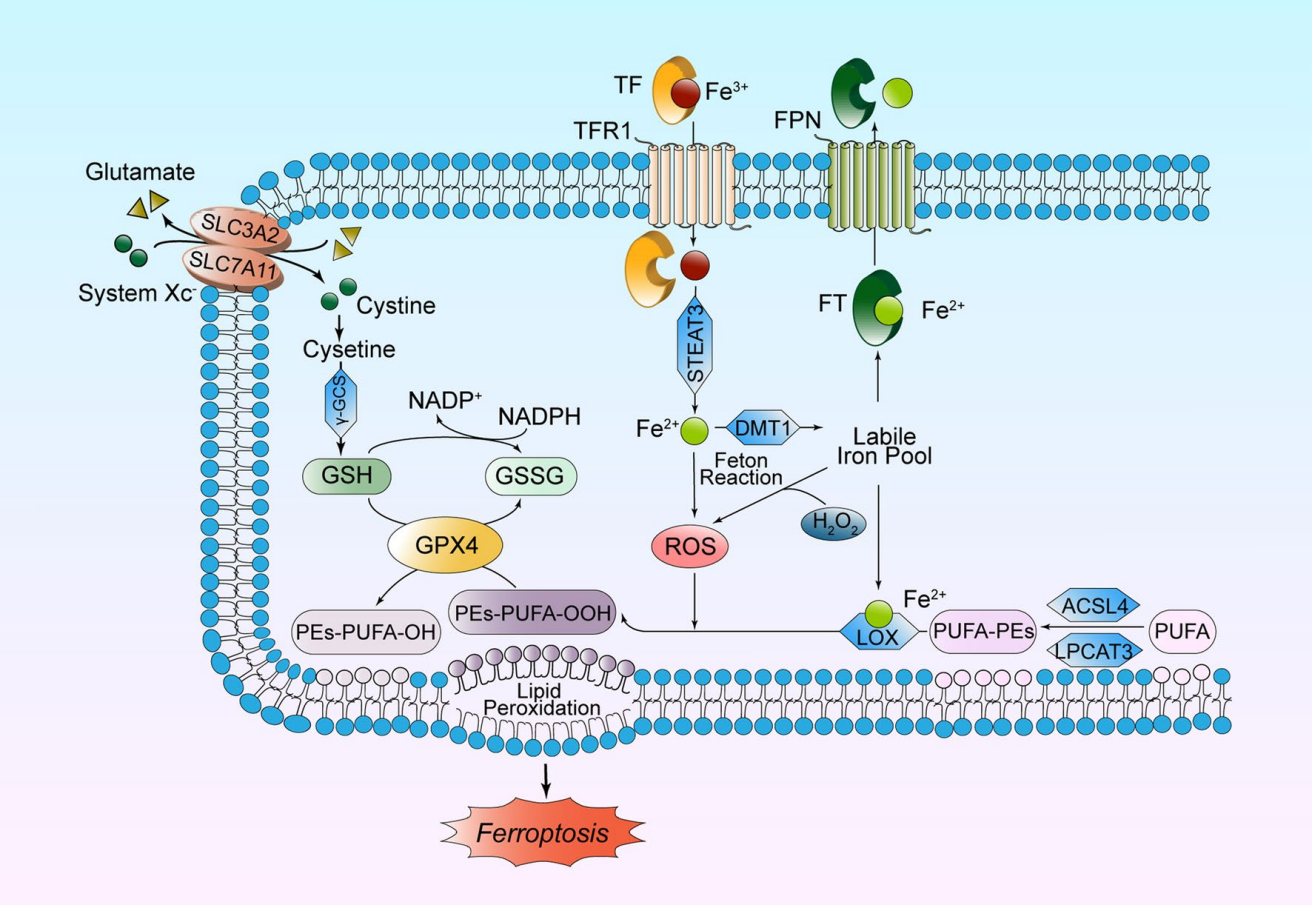
Cellular metabolic mechanism of ferroptosis. ACSL4, acyl-CoA synthetase long-chain family member 4; DMT1, divalent metal transporter 1; FPN, ferroportin; FT, ferritin; GPX4, glutathione peroxidase 4; GSH, glutathione; GSSG, oxidized glutathione; LOX, lipoxygenase; LPCAT3, lysophosphatidylcholine acyltransferase 3; NADP^+^, β-nicotinamide adenine dinucleotide phosphate; NADPH, nicotinamide adenine dinucleotide phosphate oxidase; PEs, phosphatidylethanolamines; PUFA, polyunsaturated fatty acid; ROS, reactive oxygen species; STEAT3, six-transmembrane epithelial antigen of prostate 3; TF, transferrin; TFR1, transferrin receptor 1; γ-GCS, γ-glutamyl cysteine synthetase

Within the extrinsic pathway, system Xc^−^ is involved in the metabolism of glutathione and amino acids, and FPN plays a significant role in iron metabolism. While the GPX4 of the intrinsic pathway is closely related to both the processes of lipid peroxide metabolism as well as glutathione and amino acid metabolism. These three cellular metabolic mechanisms involved in ferroptosis will be elaborated separately upon as follows (Fig. 1).

#### Iron Metabolism

As an active cofactor of many proteins, iron is necessary for oxidative metabolism, myelination and neurotransmitter synthesis in the nervous system (Jasiecki et al. 2021). Non-heme iron is absorbed into the blood circulation through the small intestine. Fe^3+^ in the plasma can bind to transferrin receptor 1 (TFR1) after loading onto the TF, which is then endocytosed into the cytoplasm. TF is mainly synthesized by the liver and encoded by the *Trf* gene, which plays an important role in transporting iron from the liver to the bone marrow and other tissues. It has been shown that hepatocyte-specific *Trf* knockout (*Trf*-KO) mice would be more susceptible to ferroptosis in the liver under a high iron diet (HID) (Yu et al. 2020). The role of TF was the result of binding to the PPRE promoter, which was inhibited by PPARα. The lack of PPARα caused a significant increase in iron transport (Xing et al. 2022). Subsequently, Fe^3+^ released from TF is reduced to Fe^2+^ by six-transmembrane epithelial antigen of prostate 3 (STEAP3) (Yan and Zhang 2019), and divalent metal transporter 1 (DMT1) transports Fe^2+^ into the labile iron pool in the cytoplasm (Zhang et al. 2021a).

On the cell membrane, FPN1, as the only protein known to transport non-heme iron out of cells, transports excessive Fe^2+^ out of cells to keep intracellular Fe^2+^ concentration within the normal range (Bu et al. 2021). A study on diabetic myocardial ischemia–reperfusion injury (IRI) showed that nuclear factor E2-related factor 2 (Nrf2) controlled the transcription of FPN1, since the Nrf2/FPN1 signal pathway was a pivotal mechanism in restricting ferroptosis (Tian et al. 2021). In addition to encoding FPN1, Nrf2 activates a series of genes with antioxidant response element (ARE) by transcription, including heme oxygenase-1 (*HO-1*), *GPX4*, *SLC7A11*. Since Nrf2 plays the roles in combination with Kelch-like ECH-associated protein 1(Keap1), the above signaling pathway is defined as Keap1/Nrf2/ARE. HO-1 induction is one of the earlier cellular responses to tissue damage and is responsible for the antioxidant and neuroprotective features of its by-products (Cornelius et al. 2013).

This strict iron metabolism pathway maintains intracellular iron homeostasis. Destruction of iron uptake, transport, storage, or utilization cause overaccumulation of iron. Excessive Fe^2+^ can react with hydrogen peroxide (H_2_O_2_) to produce and accumulate reactive oxygen species (ROS); that is, the Fenton reaction induces ferroptosis (Xu et al. 2020). The efflux Fe^2+^ is stored in FT, which is composed of the ferritin light chain 1 (FTL1) and the ferritin heavy chain 1 (FTH1), so as to prevent the formation of ROS catalyzed by H_2_O_2_ (Xie et al. 2016).

In recent years, the regulation of iron homeostasis by the iron regulatory proteins (IRPs)/iron response elements (IREs) system has attracted the attention of researchers. IRPs regulate the expression of target genes, such as *FTH1, FTL1,* and *TfR1* at the post-transcriptional level to regulate the expression of metabolic proteins associated with iron metabolism (Jeong et al. 2015; Deng et al. 2023). Hence, increasing the binding activity of IRE and IRP and the expression level of IRP1 or IRP2 improved the sensitivity to ferroptosis (Xie et al. 2016). It was found that the activation of Nrf2 significantly decreased the expression of IRP2. Thus, the cochlear hair cell damage induced by oxaliplatin could be mitigated by inhibiting ferroptosis (Xu et al. 2023a).

#### Lipid Peroxide Metabolism

The major feature of ferroptosis is the occurrence of iron-dependent LPO that preferentially occurs on polyunsaturated fatty acid (PUFA) (Panda et al. 2022). Among various subcellular structures, the endoplasmic membrane is the easiest and the first structure to undergo LPO due to it having the highest level of unsaturation. The resulting chain reaction leads to the peroxidation of the mitochondrial and plasma membranes. The breakdown of the latter is often the key link that makes cell death irreversible. PUFAs, especially arachidonic acid (AA) and adrenic acid (ADA), need to be esterified into membrane phospholipids before LPO, mainly phosphatidylethanolamines (PEs) with non-double-layer arrangement (Kagan et al. 2017). Therefore, mitochondrial membranes containing high levels of PEs play an important role in ferroptosis.

The above reaction is catalyzed by acyl-CoA synthetase long-chain family member 4 (ACSL4) and lysophosphatidylcholine acyltransferase 3 (LPCAT3). Between them, the enzymatic activity of ACSL4 cannot be replaced by other members of the ACSL family, and its overexpression promotes the sensitivity of cells to ferroptosis (Doll et al. 2017). Afterward, PEs are oxidized by lipoxygenase (LOX) to phospholipid hydroperoxide (PEs-AA/ADA-OOH), which plays a role in triggering ferroptosis (Zhang et al. 2021a; Wenzel et al. 2017). During this process, iron, as a vital component of the catalytic subunit of LOX rather than cyclooxygenase (Mao et al. 2020; Yang et al. 2016a), is involved in the production of lipid peroxides from PUFAs (Dixon and Stockwell 2014). Labile iron (not bound to enzymes) can also propagate these peroxides to drive LPO (Stockwell et al. 2020). On the other hand, ROS produced in the Fenton reaction continue to peroxide PUFA in the cell membrane, and the final products, 4-hydroxynonaldehyde and malondialdehyde, are often used as general markers of oxidative stress (Bu et al. 2021).

To fight against cell injury induced by oxidative stress and maintain dynamic oxidation–reduction homeostasis, the human body has a series of antioxidant systems. Namely, GPX4, as a phospholipid peroxidase containing selenium, is the most essential anti-lipid peroxidase in cells. Studies have shown that in the absence of GPX4, the calcium-independent phospholipase iPLA2β acts as a major ferroptosis suppressor in a GPX4-independent manner (Chen et al. 2021). In addition, there are many pathways inhibiting ferroptosis that belong to the NAD(P)H-dihydroubiquinone pathway, such as the ferroptosis-suppressor-protein 1 (FSP1) -CoQ_10_ pathway (Doll et al. 2019), the GCH1-BH_4_ pathway (Liu et al. 2023c), and the DHODH-CoQH_2_ system (Mao et al. 2021). Research showed that if the cell was attacked by ROS, Nrf2 dissociated from Keap1 and rapidly entered the nucleus, inhibiting or repairing LPO damage through the Nrf2-ARE system (Tonelli et al. 2018). When no oxidants at non-physiological concentration are added and versatile antioxidants are physiologically exhausted, cell death follows (Ratan 2020).

#### Glutathione and Amino Acid Metabolism

The mechanism of aberrant amino acid metabolism in inducing ferroptosis is indicated as such: the system Xc^−^ on the cell membrane consists of a glycosylated heavy chain SLC3A2 and a non-glycosylated light chain SLC7A11, which can exchange extracellular cystine and intracellular glutamate (Glu) in a 1:1 ratio (Yang et al. 2016b). Cystine is reduced to Cys after entering the cells, and then Cys and Glu bind with glycine (Gly) to form GSH under the catalysis of γ-glutamyl cysteine synthetase (γ-GCS), thereby exerting antioxidant effects. As a selenium-dependent GPX enzyme expressed in mammalian cells, GPX4 plays an essential inhibitory role in ferroptosis (Li et al. 2021b) (Yang et al. 2014). It converts two GSH molecules into oxidized glutathione (GSSG), and reduces lipid peroxide (PEs-AA/ADA-OOH) to the non-toxic corresponding lipid derived alcohol (PEs-AA/ADA-OH) to prevent the accumulation of harmful lipid peroxides (Maiorino et al. 2018; Ursini and Maiorino 2020).

Cys is the least abundant among the three amino acids that constitute GSH, so its input becomes the rate-limiting step of GSH de novo synthesis. Cys is also considered to be the critical cofactor for GPX4 in eliminating lipid peroxides (Stockwell et al. 2017). In addition, Cys not only participated in GPX4 synthesis, but also activates the Rag-mTORC1-4EBP axis to promote this process (Zhang et al. 2021b). Selenocysteine is the active center of GPX4. When replaced by cysteine, the activity decreased significantly and cells became more sensitive to hydrogen peroxide-induced ferroptosis (Ingold et al. 2018). It is reported that genetic deletion of LRP8, the selenoprotein receptor, leads to ferroptosis as a result of an insufficient supply of selenocysteine (Alborzinia et al. 2023). As is commonly understood, deficiency of Cys is sufficient to induce ferroptosis, however, some studies have found that when Cys availability restricts the biosynthesis of GSH, mammalian cells can also use excess methionine to synthesize Cys through the transsulfuration pathway. This method has resistance to ferroptosis induced by system Xc^−^ inhibitors (Stockwell et al. 2017).

Considering the amino acid transport mechanism of system Xc^−^, a high extracellular Glu level will suppress this transport to a certain extent and induce ferroptosis, which may also be one of the reasons why Glu accumulation in the nervous system may cause damage (Dixon et al. 2012). When the function of the system Xc^−^ is inhibited, the accumulation of Glu in the cell will promote ferroptosis through the Gln catabolic pathway (Xu, et al. 2023c). Some studies have found through metabolomics that gamma-glutamyl dipeptide or tripeptide was found in the cell under Cys deprivation. These metabolites reduced the level of Glu and alleviated the sensitivity to ferroptosis (Kagan et al. 2017). But on the other hand, for cysteine-deprived cells, the catabolism of Gln promoted the synthesis of PUFA, and its decomposition products such as α-ketoglutaric acid could also increase the accumulation of lipid peroxides. These effects of glutamine restored the sensitivity of cells to cysteine-deficient ferroptosis (Gao et al. 2019). It's also worth noting that although Gln can be decomposed to Glu catalyzed by the glutaminases GLS1 and GLS2, only the catalysis of the latter is associated with ferroptosis (Gao et al. 2015).

### Role of Ferroptosis in Ischemic Stroke

The development of ferroptosis is intensively related to ischemic brain injury, especially in neurons and microglia (Cui et al. 2021). After cerebral ischemia, the blood–brain barrier (BBB) is disrupted due to the loss of tight junctional integrity, and Fe^3+^ in the blood is released into the brain parenchyma with the assistance of TF and TFR1 (Zhao et al. 2023). Subsequently, Fe^2+^ overload occurs and is reduced and transported to the cytoplasm. So ROS, rapidly accumulated through the Fenton reaction, promotes the damage of nucleic acids, proteins and membranes, and causes ferroptosis.

When cerebral ischemia reperfusion occurs, the release of excitatory amino acids, represented by Glu, increases and accumulates in the synaptic cristae. Intracellular glutamate intake decreases while extracellular glutamate release increases, inhibiting the system Xc^−^ (Xu et al. 2023c). In short, all these metabolic imbalances caused by cerebral ischemia sharpen the accrual of ROS and the onset of ferroptosis.

**Fig. 2:**
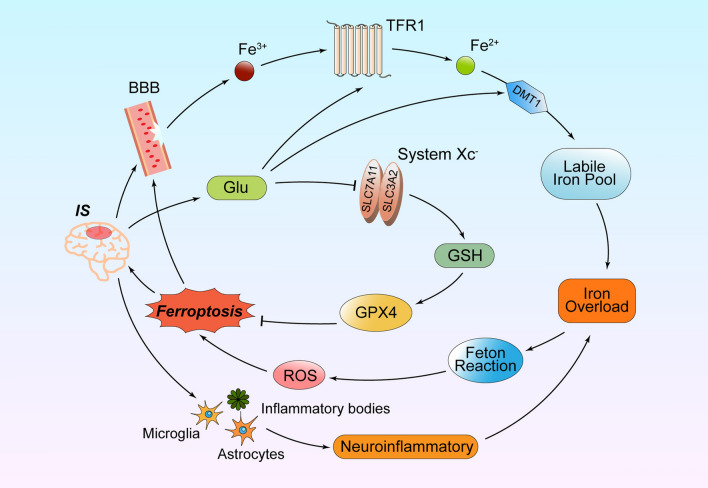
Mechanisms of ferroptosis in ischemic stroke. BBB, blood–brain barrier; DMT1, divalent metal transporter 1; Glu, glutamate; GPX4, glutathione peroxidase 4; GSH, glutathione; IS, ischemic stroke; TFR1, transferrin receptor 1

In addition, there exists a remarkable neuroinflammatory response after IS, characterized by activation of microglia and astrocytes as well as an increase in inflammatory bodies. During inflammation, activation of inflammation-related signaling pathways may contribute to ferroptosis in different ways, such as the JAK-STAT, NF-κB, cGAS-STING, and MAPK signaling pathways (Chen et al. 2023a). This phenomenon leads to increased expression of intracellular inflammatory factors, such as IL-6, IL-1β, and TNF-α, resulting in endocytosis and degradation of FPN1 regulated by hepcidin (Qian et al. 2014; Yang et al. 2020). Therefore, iron overload in cells resulted in the adverse production of excess ROS. Studies identified ACSL4 as a novel regulator of neuronal death and neuroinflammation. In tMCAO mice and microglia with OGD/R, the expression of proinflammatory cytokine IL-6, IL-1β, and TNF-α were reduced with the knockdown of ACSL4, which confirmed that ACSL4 could promote microglia-mediated inflammatory response (Cui et al. 2021) (Fig. 2).

In conclusion, IS induces ferroptosis, which in turn aggravates IS. Therefore, designing drugs to target ferroptosis for the treatment of IS holds great prospect.

## Therapeutic Targets for Ferroptosis in Ischemic Stroke

### GPX4

GPX4 is a phospholipid peroxidase containing selenium and is the most critical anti-lipid peroxidase in the cell, and the GPX4-GSH-Cys axis is the central pathway to inhibiting ferroptosis. Up-regulation of GPX4 was proven to depress ferroptosis in IS or HS, while excessive and lethal accumulation of lipid ROS in biofilms led to the depletion of GSH and inactivation of GPX4 (Jin et al. 2021).

Alim et al. found in the mouse model of IS and HS that pharmacological selenium (Se) supplementation could augment GPX4 and other genes in transcriptional programming to protect neurons by co-activating transcription factors TFAP2c and Sp1. In the rodent model of focal IS, intraperitoneal injection of selenocysteine-containing peptide, Tat-SelPep, drove the expression of antioxidant GPX4 to resist ROS, so that ferroptosis and oxidative stress were countered and cerebral infarct size was diminished (Alim et al. 2019). In addition, docosahexaenoic acid (DHA) that is rich in neuronal membrane phospholipids could modulate GPX4 gene expression by up-regulating the cytoplasmic intron sequence-retaining GPX4 Cytoplasmic Intron-sequence Retaining Transcripts (CIRT), thereby improving neuronal antioxidant capacity (Zhang et al. 2021a). Dopamine, a critical neurotransmitter, inhibited the degradation of dopamine receptors DRD4 and GPX4 in erastin-induced ferroptosis, thus reducing the development of ferroptosis (Wang et al. 2016). Levodopa treatment was approved for patients with early or late-stage stroke (Wang et al. 2016). The compound carvacrol repressed ferroptosis by increasing GPX4 expression, thus preserving gerbil hippocampal neurons from cerebral IRI (Guan et al. 2019).

By exploring the mechanisms underlying the changes in GPX4 activity, it is revealed that GPX4 up-regulation is strictly mediated by intracellular signaling pathways. GPX4 is a transcriptional target of Nrf2, so the Nrf2 signaling pathway will directly or indirectly affect the activity of GPX4 (Dodson et al. 2019). Studies focused on the CDGSH iron-sulfur domain 2 (CISD2), a member of the iron-sulfur cluster protein family, suggested that overexpression of CISD2 protected the antioxidant system from IS injury by activating the Nrf2/HO-1 signaling pathway in mice (Hu et al. 2023). Salvia miltiorrhiza polysaccharide 1 (SMP1) protected PC12 cells from OGD/R-induced ferroptosis and LPO by activating the Nrf2/HO-1 pathway (Meng et al. 2022). Similarly, β-caryophyllene (BCP) remarkably enhanced Nrf2 nuclear translocation, so as to activate the Nrf2/HO-1 pathway and elevate GPX4 expression. When OGD/R-induced ROS production and iron accumulation were lessened, the occurrence of ferroptosis was prevented. This neuroprotective effect of BCP could be reversed by ML385 (an Nrf2 inhibitor) (Hu et al. 2022).

Other compounds including 15, 16-Dihydrotanshinone I (Wu et al. 2023), ferrostatin-1 analog Srs11-92 (Chen et al. 2023b), Loureirin C (Liu et al. 2023d), Astragaloside IV (Zhang et al. 2023), Vitexin (Guo and Shi 2023), propofol (Fan et al. 2023), may play a therapeutic role through this pathway. What’s more, Nrf can compensate for the effect of GSH-GPX4 by upregulating the thioredoxin system. Many detoxifying enzymes that target the downstream products of peroxidation after ferroptosis are also activated by Nrf2. In general, using these compounds is expected to be a new pathway to developing drugs for IS by suppressing ferroptosis.

### System Xc^−^

Cystine/Glu exchanger xCT (SLC7A11) is a functional subunit of the system Xc^−^ amino-acidantiporter. This transport system contains a 1-pass transmembrane regulatory subunit (SLC3A2), which is connected by a disulfide bond (Ratan 2020). The dysfunction of system Xc^−^ can be initiated by the inactivation of its particular subunit xCT (SLC7A11) as a functional subunit (Pampliega et al. 2011).

Erastin, a specific small-molecule inducer of ferroptosis, was named in the work where this non-apoptotic mode of cell death was initially reported. Subsequent studies gradually confirmed that erastin, the first inhibitor of system Xc^−^, was able to hinder cystine intake by inactivating SLC7A11 function. Currently, it is shown that erastin analogues, such as sulfasalazine, sorafenib, glutamate, and lanperisone (Bu et al. 2021), could all repress system Xc^−^ and thereby induce ferroptosis. Among them, imidazole ketone erastin (IKE) had a stronger inhibitory effect and greater metabolic stability than erastin, which led IKE to be an excellent candidate for investigating ferroptosis initiation in vivo and in vitro (Hirschhorn and Stockwell 2019). Recent studies have also confirmed that focal cerebral ischemia–reperfusion led to extensive changes in the expression of miRNAs in the rat brain, especially miR-27a. Further research showed that microRNA-27a may induce ferroptosis and aggravate MCAO/R injury in rats by down-regulating SLC7A11 (Zhu et al. 2023).

The activation of some signaling molecules will eventually play a vital part through SLC7A11, as demonstrated in the study on protective strategies against oxidative stress and ferroptosis in patients with cognitive impairment after IS. At the mRNA level, two major transcription factors have been identified that regulate stress-induced transcription of *SLC7A11*, namely, Nrf2 and ATF4 (Pakos-Zebrucka et al. 2016). Therefore, the activation of Nrf2 would increase the expression of SLC7A11 (Koppula et al. 2018) and make differences in resisting oxidative stress and ferroptosis during cerebral ischemia through the signaling pathway jointly participated by SLC7A11/GPX4. Rehmannioside A ameliorated cognitive impairment and alleviated ferroptosis by activating the PI3K/Nrf2/SLC7A11 signaling pathway (Fu et al. 2022). Kaempferol reversed OGD/R-induced ferroptosis in primary mouse cortical neurons by activating the Nrf2/SLC7A11/GPX4 axis (Yuan et al. 2021b). In addition, some studies have indicated that the effect of the compound on inhibiting ferroptosis is related to SLC7A11, but the specific regulatory mechanism is not clear. Dl-3-n-butylphthalide played neuroprotective roles in MCAO/R rats by ferroptosis regulation via the SLC7A11/GSH/GPX4 pathway (Xu et al. 2023b). NTE (extract of Naotaifang, a compound Chinese herbal medicine) also inhibited ferroptosis in MCAO rats through the TFR1/DMT1 and SLC7A11/GPX4 pathways (Lan et al. 2020).

The effects of Glu analogues and extracellular Glu concentration on ferroptosis are not absolute. Bannai et al. documented that long-chain Glu analogues (such as quinoxaquine) could potently dampen the transport of cystine, while short-chain Glu analogues (such as aspartic acid or N-methyl-D-aspartate (NMDA)) exhibited no effect (Bannai 1986). More significantly, during IRI, extracellular Glu is often in excess, leading to inhibition of the system Xc^−^ fuction (Dixon 2017). However, this does not mean that promotion of system Xc^−^ will certainly suppress ferroptosis. It was reported that elevated expression of xCT also stimulated Glu release (Pampliega et al. 2011) and induced long-lasting glutamate excitotoxicity in the rat model of cerebral ischemia–reperfusion (Hsieh et al. 2017). In other words, when the up-regulation of system Xc^−^ reaches a certain threshold, Glu toxicity and ferroptosis will be aggravated. This complex function of system Xc^−^ occasionally depends on the type of nerve cells. For instance, system Xc^−^ was up-regulated in astrocytes and microglia of the rat model suffering stroke, while its inhibition attenuated neuroinflammatory and IRI (Domercq et al. 2016). On the contrary, suppression of Glu on system Xc^−^ facilitated ferroptosis of primary oligodendrocytes by activating acid sphingomyelinase (Domercq et al. 2016).

### ACSL4

In addition to lipid peroxidases, several genes that modulate PUFA synthesis and maintain normal cell membranes integrity may also affect the occurrence of ferroptosis. ACSL4 is often regarded as a driving factor of ferroptosis by enhancing the induction effects of erastin and RSL3 (Shintoku et al. 2017), and its expression also determines the susceptibility of the cell to ferroptosis (Fang et al. 2022).

The experimental results indicated that forced overexpression of ACSL4 exacerbated ischemic brain injury in mice, while knockout exerted a beneficial effect (Cui et al. 2021). For instance, it was found that IS would lead to elevated ACSL4 and 15-LOX-2 protein levels, which had never been reported before (Li, et al. 2021a). This overexpression of ACSL4 was controlled by miR-347, which increased after IS and up-regulated ACSL4 at the transcriptional or post-transcriptional levels (Gubern et al. 2013). Additionally, specific protein 1 (Sp1) was also considered as a crucial transcription element to facilitate ACSL4 expression by binding to the promoter region of ACSL4 (Li et al. 2019).

Elevated expression of ACSL4 aggravates IS by contributing to ferroptosis-induced cerebral injury and neuroinflammation. ACSL4 can enhance LPO to stimulate neuronal death (Cui et al. 2021), while knockout of the *Acsl4* gene inhibits the accumulation of LPO substrates and grants cells obvious resistance to ferroptosis (Doll et al. 2017). On the other hand, post-ischemic inflammation seems to be involved in all stages of cerebral IRI. In this process, microglia are considered important participants, and knockdown of ACSL4 can restrain the production of proinflammatory cytokines in microglia, such as TNFα, IL-6, and IL-1β (Cui et al. 2021).

However, the relationship between cerebral ischemia and ACSL4 expression cannot be generalized. It was proven that the expression of ACSL4 would be suppressed in the early stage of IS, and this inhibition was induced by HIF-1α. The increase of HIF-1α protein was also observed in mice and primate brains after experimental cerebral ischemia (Speer et al. 2013), which might account for the early neuroprotective role of HIF-1α (Cui et al. 2021). As shown in a recent study, enriched environment (EE) promoted HIF-1α expression and thus inhibited ACSL4 expression at the transcriptional level, ultimately acting to inhibit ferroptosis in MCAO/R rats (Liu et al. 2023b).

We previously mentioned that the repression of ACSL4 expression would cause significant protection against ferroptosis. Thiazolidinediones (TZDs) are a class of insulin sensitizers recommended for the treatment of type 2 diabetes, and have been confirmed to specifically inhibit ACSL4 activity and prevent ferroptosis (Doll et al. 2017). These include rosiglitazone (ROSI), troglitazone (TRO), pioglitazone (PIO). It was demonstrated that PIO could decrease infarct volume and improve the neurological score of transient MCAO mice (Jin et al. 2021). Reduction in infarct volume and inflammation as well as improvement in neurological function were observed during the treatment with PIO of rats before or after MCAO (Liu et al. 2023b). As a synthetic agonist of proliferation-activated receptor γ (PPARγ), TZDs exhibited no correlation between their anti-ferroptosis ability and PPAR-γ-mediated gene transcription (Doll et al. 2017). This effect might be achieved by the fact that chromanol rings endow the tocopherols with antioxidant activity (Zhang et al. 2021a). That is, TZDs restrained ACSL4 to block the enzymatic reaction during LPO, thus interfering with ferroptosis in vivo and in vitro (Doll et al. 2017). Calycosin is a substance that is able to play a neuroprotective and antioxidant role in cerebral ischemia/reperfusion injury. Recent studies have found that calycosin inhibited tMCAO/R or OGD/R-induced ACSL4 upregulation and promoted the recovery of neural function after cerebral ischemia in rats (Liu et al. 2023a). With relatively well-defined mechanism research as a foundation, ACSL4 has become a critical target to prevent ferroptosis in IS.

### LOX

LOX is a key enzyme that catalyzes PEs to produce lipid peroxides, and iron is also a vital component of lipoxygenase catalytic subunits. Because of their high homology, 12-LOX and 15-LOX-1 are collectively referred to as 12/15-LOX. Generally speaking, free PUFAs are the preferred substrate of LOX, but PUFA-containing phospholipids (PUFA-PEs) are not. However, when PUFA acts as a ferroptotic signal, PEs can form a non-bilayer arrangement (Dolma et al. 2003), promoting PUFA-PEs rather than free PUFA to generate LPO through LOXs (Stockwell et al. 2017). It is noteworthy that 12/15-LOX is special. It can directly oxidize lipid membranes containing PUFA without phospholipase, causing the straightforward attack on organelles including mitochondria (van Leyen et al. 2014).

Earlier studies evidenced that 12/15-LOX was overexpressed under pathological conditions in humans and mice after stroke (van Leyen et al. 2006; Yigitkanli et al. 2013). A recent work also showed that 12/15-LOX was highly expressed in the pMCAO mouse model (Karatas et al. 2018), and its elevated expression led to neuronal death and BBB destruction. Silencing the arachidonate lipoxygenase (*Alox*) genes made cells resistant to erastin-induced ferroptosis (Yang et al. 2016a). When 12/15-LOX in mouse neurons was knocked out, cerebral ischemia injury was avoided (Tuo et al. 2022). Not only that, 12/15-LOX inhibitors could improve neuronal damage and reduce edema and infarct size. These results indicate that 12/15-LOX can be regarded as an effective target for stroke treatment (Panda et al. 2022). Furthermore, it has been found that 5-LOX, another subtype of LOX, is also an essential target in repressing ferroptosis, as it has the ability to produce toxic lipids and induce ferroptosis (Zhang et al. 2021a).

At present, the suppression of LOX has been reflected in compounds under development or in marketed drugs. Zileuton, as a selective inhibitor of 5-LOX, is a potent radical-trapping antioxidant (RTA) (Xie et al. 2016). The current research results of 15-LOX-1 inhibitors are more extensive. For example, baicalein was previously proven to be a 12/15-LOX inhibitor with a protective effect on cerebral IRI. A recent investigation profoundly revealed that baicalein could lessen the levels of iron and LPO products in the brain tissue of tMCAO mice, enhance the morphological features of ferroptosis, and significantly alleviate cerebral IRI (Li et al. 2022a). This study further confirmed that baicalein had repressive activity on RSL3-induced ferroptosis in HT22 cells. What’s more, the researchers discovered ML351, a novel chemical inhibitor of 12/15-LOX, through high throughput screening. In the mice model of ischemic brain injury, ML351 combined with tPA relieved BBB destruction and neurologic impairment. The above effect occurred due to the fact that this treatment inhibited 12/15-LOX and activated the JNK signaling pathway to reverse the substantial increase of LPO product 12-HETE (Cheng et al. 2021). At the same time, ML351 not only has good nanoscale titer and higher IC_50_, but also has better selectivity for 12/15-LOX than other isoenzymes (Rai et al. 2010). Additionally, 12/15-LOX inhibitor nordihydroguaiaretic acid (NDGA) also had a similar dampening effect (Probst et al. 2017). Many other inhibitors (such as ML351, LOX Block-1, BW-B 70C, PD146176, U0126, curcumin, and vitamin E family members) have also been demonstrated to remarkably attenuate cerebral injury after IS regulated by ferroptosis (Dixon et al. 2012; Stockwell et al. 2017; Magtanong and Dixon 2018).

### Others

Proteins associated with iron metabolism are key factors in ferroptosis. Recent studies on IS showed that certain factors, such as iron deficiency and the use of oral contraceptives, are risk factors of thromboembolic diseases by upregulating TF (Tang et al. 2020a). Administration of TF antibodies or peptides to interfere with TF knockout could significantly reduce the incidence of IS (Tang et al. 2020b). Moreover, FSP1 is a component of the FSP1/CoQ_10_/NADH system that compensates for GPX4-deficient enzyme-specific catalytic systems. In rat model of IS compound 3f acted as a specific ferroptosis inhibitor by increasing FSP1 protein levels and effectively alleviating brain injury (Fang et al. 2022).

*p53* is a tumor suppressor gene involved in ferroptosis as a transcription factor for SLC7A11. It was found that miR-214 can reduce the level of p53 through the lncRNA PVT1/miR-214/p53 positive feedback loop, thus significantly inhibiting ferroptosis in mice by increasing the amino acid transport of SLC7A11 (Lu and Xu 2020). Therefore, the regulation of p53 is also an important pathway in affecting ferroptosis.

A growing number of works have reported that there is a certain correlation between ferroptosis and autophagy, another mode of PCD. During autophagy, nuclear receptor coactivator 4 (NCOA4) selectively binds to FT and transfers it to lysosomes for degradation, causing an increase in labile iron and thereby resulting in ferroptosis (Stockwell et al. 2020). Compound 9a notably ameliorated ischemic reperfusion injury by repressing NCOA4 and diminishing the amount of bioavailable Fe^2+^ in cells, hence preventing the interaction between NCOA4 protein and FTH1 (Fang et al. 2021).

Hormesis is a new concept that can be applied to neurological disorders in which oxidative stress is involved and been closely related with ferroptosis, such as IS and some neurodegenerative diseases (Cosentino et al. 2023). Hormesis is featured with biphasic dose response, meaning that low doses of the stressor are neuroprotective, while high doses can exert a neurotoxic action in the brain. There are large numbers of agents that have been shown to induce hormesis, and this phenomenon is also applicable in many plant-derived agents that are both part of normal diets and widely used as nutrient supplements (called “hormetic nutrients”), such as polyphenols (Modafferi et al. 2023) and flavonoids (Li et al. 2023). Some of hormetic nutrients play neuroprotective roles depending on the appropriate dose by activating Nrf2. For instance, cariside II (a naturally occurring flavonoid derived from Herba Epimedii) preconditioning evoked robust neuroprotection against IS by targeting Nrf2 and OXPHOS/NF-κB/ferroptosis pathways (Gao et al. 2023). Preconditioning avenanthramide-C activated the Nrf2/ARE pathway by inhibiting the ferroptosis to improve cognitive dysfunction (Ma et al. 2023). Soybean isoflavones preconditioning reduced cerebral IRI by inhibiting ferroptosis and inflammatory response and protecting the BBB (Scuto et al. 2022).

In fact, the above effects can be summarized as the result of a combination of exogenous antioxidants and endogenous cellular defense mechanisms (Calabrese et al. 2010). Modulation of the latter represents an innovative approach to therapeutic intervention in diseases. For example, knowledge of the endogenous physiological actions of NO in the nervous system raises the possibility of manipulating the NO system for therapeutic benefit (Calabrese et al. 2007). In addition, a part of cellular stress responses is reflected in producing active molecules endowed with antioxidant activity encoded by cytoprotective genes, called vitagenes (Calabrese et al. 2016). Examples of these molecules include HO-1, bilirubin, thioredoxin, and thioredoxin reductase, all of which can be upregulated by Nrf2/ARE (Calabrese et al. 2010). Curcumin, an exogenous adjuvant, alleviated neurodegeneration and related diseases through the Nrf2 and vitagenes, mainly the HO-1 (Concetta et al. 2019). Moreover, under the catalysis of HO-1, heme could eventually be catabolized to bilirubin, which was confirmed to be an endogenous cytoprotective molecule because of its ability to scavenge peroxyl radicals (Calabrese et al. 2016; Mancuso et al. 2008). In conclusion, the neuroprotective effects of hormetic nutrients are closely related to vitagene network. Therefore, appropriate doses of these agents are expected to alleviate ferroptosis in CNS diseases through the Nrf2 and vitagenes pathways (Table 1).

**Table 1:** Compounds targeting ferroptosis for the treatment of IS

Target	Reagent	Impact on ferroptosis	Therapeutic effect on IS	Reference
GPX4	DHA	Up-regulates the expression of the GPX4	Prevents neurological and vascular disorders induced by IS	Zhang et al. 2021a
	Tat-SelPep	Drives the expression of antioxidant GPX4 to resist ROS	Reduces infarct volume following focal ischemia	Alim et al. 2019
	Levodopa	Reduces the degradation of GPX4	Reorganizes neuronal networks in the ischemic territory	Wang et al. 2016
	carvacrol	Increases GPX4 expression	Prevents hippocampal neuron damage and cognitive dysfunction	Guan et al. 2019
	SMP1	Activates Nrf2/HO-1 pathway and increasing GPX4 expression	Protects cells and animals from oxidative stress injury associated with IS	Meng et al. 2022
	BCP	Activates Nrf2/HO-1 pathway and increasing GPX4 expression	Exerts anti-oxidative stress and neuroprotective effect	Hu et al. 2022
System Xc^−^	Rehmannioside A	Activates the PI3K/Nrf2/SLC7A11 signaling pathway	Has neuroprotection effect and improves cognitive impairment after IS	Fu et al. 2022
	Dl-3-n-butylphthalide	Activates the SLC7A11/GSH/GPX4 pathway	Addresses psychiatric and behavioral functions following acute IS	Xu et al. 2023c, a, b
	kaempferol	Activates the Nrf2/SLC7A11/GPX4 axis	Has the protective effects on antioxidant capacity and lipid peroxidation	Yuan et al. 2021b
	NTE	Activates TFR1/DMT1 and SLC7A11/GPX4 pathways	Plays a neuroprotective role and improves neurological dysfunction	Lan et al. 2020
ACSL4	TZDs	Inhibits ACSL4 activity specially	Reduces infarct volume and inflammation as well as improve neurological function	Doll et al. 2017
	calycosin	Inhibits the upregulation of ACSL4	Promotes the recovery of neural function	Liu, et al. 2023a
LOX	Zileuton	Selective inhibitor of 5-LOX and effective radical-trapping antioxidant	Reduces inflammatory reaction and ischemic brain damage	Xie et al. 2016
	baicalein	Inhibiting 12/15-LOX and reducing the levels of iron and LPO products	Exerts anti-oxidative stress and neuroprotective effect	Li et al. 2022a
	NDGA	Inhibiting 12/15-LOX and reducing the levels of iron and LPO products	Exerts anti-oxidative stress and neuroprotective effect	Probst et al. 2017
Iron	compound 9a	Inhibiting NCOA4 and reducing the amount of bioavailable Fe^2+^ in cells	Protects brain from IRI	Fang et al. 2021

## Summary and Prospect

Although numerous studies have clarified the pathophysiological process of stroke, there are still new pathogenesis and possible therapeutic targets emerging one after another, including ferroptosis. We summarized the cellular metabolic mechanism of ferroptosis and expounded on the feasibility of suppressing ferroptosis as a therapeutic target for IS. In this review, we aimed to provide insights into more feasible treatment schemes in the process of iron, amino acid, and lipid peroxide metabolism based on the currently proven effective compounds and drugs.

Though the discovery of ferroptosis at the beginning of this century sparked much research and discussion, the application of ferroptosis in treating IS is still a lengthy process. From the perspective of the pathogenesis of ferroptosis, in addition to abnormal metabolism of iron, amino acids, and lipid peroxides, several signaling pathways are involved in modulating ferroptosis, such as the p53-SAT1-ALOX15 pathway (Ou et al. 2016), p53/SLC7A11 pathway (Chen et al. 2021), p62-Keap1-Nrf2 pathway (Li et al. 2022b), FSP1-CoQ_10_-NAD(P)H pathway (Chen et al. 2022). The range of these signaling pathways is constantly updated and expanded upon, and they may have complex intersections and interactions. Moreover, we also mentioned that the levels of some key molecules or proteins did not regulate the progression of ferroptosis in a unidirectional manner. Therefore, it is difficulty to explore targeted inhibitors because of the partially ambiguous mechanism of ferroptosis, and it is likely that developing a single inhibitor will not achieve high efficacy. Multiple modes of cell death such as ferroptosis, autophagy, apoptosis, and necrosis are often present simultaneously. In different cells and different types of cerebral ischemia, identifying the aspect of focus also emerges as an important problem hindering drug design.

From the translation of preclinical evaluation to clinical trial, model animals for IS are relatively simple and cannot fully represent the more complex pathological features of human beings. At present, researches on ferroptosis focuses on four main aspects including morphological characteristic detection, gene expression detection, protein level detection and biochemical characteristic index detection. Consequently, the lack of a clear and authoritative criteria for ferroptosis detection has become a pressing issue in preclinical and clinical trials. Moreover, because of the essential role of iron ions and free radicals in life, the toxicity of iron chelators, free radical capture inhibitors and other drugs has greatly affected their pharmaceutical properties in clinical trials. Currently, hormetic nutrients and agents modulating vitagenes are also drawing increasing attention of inhibiting ferroptosis due to their protection against oxidative stress and neuroinflammation in CNS diseases. However, it is still a problem that requires attention to grasp the safety and efficacy of specific doses. Due to the complexity of ferroptosis, there remains a long road to treating IS.

## Data Availability

Data sharing is not applicable to this article as no datasets were generated or analyzed during the current study.

## Abbreviations

AA: Arachidonic acid
ACSL4: Acyl-CoA synthetase long-chain family member 4
ADA: Adrenic acid
ALOX: Arachidonate lipoxygenase
ARE: Antioxidant response element
BBB: Blood–brain barrier
BCP: β-Caryophyllene
BH_4_: Tetrahydrobiopterin
CIRT: Cytoplasmic Intron-sequence Retaining Transcripts
CISD2: CDGSH iron-sulfur domain 2
CNS: Central nervous system
CoQ_10_: Coenzyme Q_10_
Cys: Cysteine
DHA: Docosahexaenoic acid
DMT1: Divalent metal transporter 1
EE: Enriched environment
FPN: Ferroportin
FSP1: Ferroptosis suppressor protein 1
FT: Ferritin
FTH1: Ferritin heavy chain 1
FTL1: Ferritin light chain 1
GCH1: Cyclohydrolase 1
Gln: Glutamine
Glu: Glutamate
Gly: Glycine
GPX4: Glutathione peroxidase 4
GSH: Glutathione
GSSG: Oxidized glutathione
HID: High iron diet
H_2_O_2_: Hydrogen peroxide
HO-1: Heme oxygenase-1
HO·: Hydroxyl radical
HS: Hemorrhagic stroke
IKE: Imidazole ketone erastin
IREs: Iron response elements
IRI: Ischemia–reperfusion injury
IRPs: Iron regulatory proteins
IS: Ischemic stroke
Keap1: Kelch-like ECH-associated protein 1
LOX: Lipoxygenase
LPCAT3: Lysophosphatidylcholine acyltransferase 3
LPO: Lipid peroxidation
LTF: Lactotransferrin
MCAO: Middle cerebral artery occlusion
NCOA4: Nuclear receptor coactivator 4
NDGA: Nordihydroguaiaretic acid
NMDA: N-methyl-D-aspartate
Nrf2: Nuclear factor E2-related factor 2
OGD: Oxygen glucose deprivation
PCD: Programmed cell death
PEs: Phosphatidylethanolamines
PIO: Pioglitazone
PPARγ: Peroxisome proliferators-activated receptor γ
PUFA: Polyunsaturated fatty acid
PUFA-PEs: PUFA-containing phospholipids
ROS: Reactive oxygen species
ROSI: Rosiglitazone
RTA: Radical-trapping antioxidant
SAH: Subarachnoid hemorrhage
Se: Selenium
Ser: Serine
SMP1: Salvia miltiorrhiza polysaccharide 1
SODs: Superoxide dismutases
Sp1: Specific protein 1
STEAT3: Six-transmembrane epithelial antigen of prostate 3
TF: Transferrin
TFR1: Transferrin receptor 1
TRO: Troglitazone
TZDs: Thiazolidinediones
γ-GCS: γ-Glutamyl cysteine synthetase
